# Advances in development of biomarkers for brain damage and ischemia

**DOI:** 10.1007/s11033-024-09708-x

**Published:** 2024-07-13

**Authors:** Diana Karimova, Elham Rostami, Vladimir N. Chubarev, Vadim V. Tarasov, Helgi B. Schiöth, Mathias Rask-Andersen

**Affiliations:** 1 Functional Pharmacology and Neuroscience, Department of Surgical Sciences, Uppsala, University, Uppsala, Sweden; 2 Department of Medical Sciences, Section of Neurosurgery, Uppsala University, Uppsala, Sweden; 3 Advanced Molecular Technology, Limited Liable Company (LLC), Moscow, 354340 Russia; 4 Department of Immunology, Genetics and Pathology, Science for Life Laboratory, Uppsala University, Uppsala, Sweden

**Keywords:** Biomarkers, Brain damage, Ischemia, Clinical trials

## Abstract

Acquired brain injury is an urgent situation that requires rapid diagnosis and treatment. Magnetic resonance imaging (MRI) and computed tomography (CT) are required for accurate diagnosis. However, these methods are costly and require substantial infrastructure and specialized staff. Circulatory biomarkers of acute brain injury may help in the management of patients with acute cerebrovascular events and prevent poor outcome and mortality. The purpose of this review is to provide an overview of the development of potential biomarkers of brain damage to increase diagnostic possibilities. For this purpose, we searched the PubMed database of studies on the diagnostic potential of brain injury biomarkers. We also accessed information from Clinicaltrials.gov to identify any clinical trials of biomarker measurements for the diagnosis of brain damage. In total, we present 41 proteins, enzymes and hormones that have been considered as biomarkers for brain injury, of which 20 have been studied in clinical trials. Several microRNAs have also emerged as potential clinical biomarkers for early diagnosis. Combining multiple biomarkers in a panel, along with other parameters, is yielding promising outcomes.

## Introduction

According to the WHO, millions of people are diagnosed with acquired brain injury every year. There were 6.2 million deaths from stroke in 2019, which makes this the 2nd highest cause of death. In addition, 1.8 million cases of neurodegenerative disease is reported every year [1]. Diseases caused by brain pathology can be prevented with population-based screening, which allows detection of brain damage at earlier stages, when the prognosis for curative treatment is significantly higher compared to later stages.

The central nervous system (CNS) is very sensitive to any type of hypoxia. Lack of oxygen leads to biochemical, structural and functional changes. Brain damage due to stroke, hemorrhage, transient ischemic attack (TIA) or traumatic brain injury (TBI), can lead to cerebral hypoxia and neuronal destruction. Brain ischemia is an urgent situation which should be solved as fast as possible. Once hypoxia is triggered, it goes through certain stages: First, bioenergetic failure develops and ion homeostasis is disrupted. Then, a free interval takes place where the bioenergetic state is restored for a while and synaptic activity returns. Finally, secondary cell death takes place, during which the metabolic rate and blood flow can be depressed. Lack of oxygen leads also to ischemic acidosis [2].

Currently, Magnetic resonance imaging (MRI) and Computed tomography (CT) are the most frequently used screening methods for diagnosis of brain damage. However, these methods expose patients to radiation, have high costs and are unable to identify early stage pathologies. It would be helpful to have effective, non-invasive tests to screen patients who are at risk of brain damage and detect this pathology in early stages.

Biomarkers are molecules measured in plasma, serum or other body fluids to indicate disease. A biomarker should be easy to identify, non-invasive, and have good sensitivity and specificity. Blood markers of acute brain injury may help in the management of patients with acute cerebrovascular events. The aim of this review is to highlight potential biomarkers for brain damage and to present recent findings on biomarkers for different types of brain damage.

## Approach

We searched the PubMed database for studies on potential biomarkers for brain damage. We used the search terms: “markers”, “serum”, “brain damage”, “cerebral damage”, “brain hypoxia”, “cerebral hypoxia”, “brain ischemia”, “cerebral ischemia” and “stroke”. The papers were filtered for studies specifically on biomarkers for brain damage. We identified 120 original research articles on 38 specific molecules that have been considered as potential brain damage biomarkers. Markers were classified into four broad categories for clarity of presentation based on the potential markers’ biochemical structure. Markers from different groups are also described in accordance with the principle of their clinical usage in different stages of brain ischemia, and combination into panels for increasing their diagnostic potential. In total, we identify six enzymes, four hormones, 14 proteins and 17 nucleic acids (13 small non-coding and four long non-coding RNAs) that have been proposed as biomarkers for brain damage (Tables 1, 2, 3 and 4). Markers that were identified in the literature were also queried in clinicaltrials.gov to identify any clinical trials that utilize these biomarkers for diagnosis of brain damage. Out of the 41 identified markers, 20 have been explored as biomarkers for brain damage in clinical trials (Tables 1, 2, 3 and 4).

**Table 1:** Enzyme biomarkers for brain damage. Information taken from Uniprot.org. Process stage column may be useful in using a marker at a certain stage of the brain damage. Mass - The mass of the biomarker in kDa. Length – the length of peptide in amino acids

Marker	Synonyms	Functions	Mass (kDa)	Length (amino acids)	UniProt ID	Damage type	Process stage	Grouping for diagnostic	Clinical trials
NDKA	Nucleoside diphosphate kinase A	Transfers phosphate groups from ATP to other nucleoside diphosphates.	17.1	152	P15531	ischemia	Acute stage, 3 h after stroke	No	NCT01833312
PARK7	Parkinson disease protein 7, Protein/nucleic acid deglycase DJ-1	Multifunctional protein, which plays a role in cell protection against oxidative stress and cell death	19.9	189	Q99497	ischemia	acute stage, 3 h after stroke	No	NCT02408562
tPA	PLAT; T-PA; TPA; plasminogen activator, tissue type	Converts the inactive, zymogen plasminogen to plasmin by hydrolyzing a single Arg-Val bond in plasminogen.	62.9	562	P00750	ischemia (because of thrombosis)	acute stage (breakdown of blood clots, fibrinolysis)	H-FABP, tPA, PAI-1, and IL-6. Also, as independent predictor of stroke.	NCT00147316 NCT02448069 NCT00147316 NCT02448069
MMP9	92 kDa type IV collagenase, 92 kDa gelatinase or gelatinase B	Degrades collagen to regulate the extracellular matrix.	78.5	707	P14780	ischemia, inflammation	regulates remodeling processes	No	NCT01926834 NCT03733223
NSE	Gamma-enolase, enolase 2 (ENO2)	Catalyzes the conversion of 2-phospho-D-glycerate to phosphoenolpyruvate PEP in the glycolytic pathway, and the reverse reaction in the gluconeogenesis pathway (PMID: 26,530,364)	47.3	434	P09104	ischemia, neuronal intracellular damage, inflammation	72 h after	No	NCT03062566 NCT04602325 NCT04266665 NCT00490295 NCT06327776
CKBB	BB-CK, Creatine kinase B chain, Brain creatine kinase, Creatine phosphokinase B-type	Catalyzes the transfer of phosphate between ATP and various phosphogens (e.g., creatine phosphate), Kinase, Transferase	42.6	381	P12277	intracellular damage, stroke, comes from the cytoplasm of destroyed cells to extracellular fluid	48–72 h	CRP, CKBB	NCT01428401 NCT03899532 NCT03927872

**Table 2:** Hormone markers for brain damage. ID numbers in databases: ^a^- UniProt ID, ^b^ – CHEBI ID. Mass - the mass of the biomarker in kDa. Length – the length of peptide in amino acids

Marker	Alternative names	Mass, kDa	Length (amino acids)	Coding gene	Reference ID	Receptor	Clinical trials
**BNP**	Ventricular natriuretic peptide; brain natriuretic peptide	14.726	134	NPPB	P16860^a^	Natriuretic peptide receptor-A, B (NPR-A, NPR-B)	NCT03377465 NCT03275155 NCT02142413
**T3**	Triiodothyronine	650.977 g/mol	20	DIO2	18,258^b^	Thyroid hormone receptor	No
**T4**	Thyroxine	776.87 g/mol	21	TG, TPO	30,660^b^	Thyroid hormone receptor	No
**TSH**	Thyroid-stimulating hormone, Thyrotropin-releasing hormone (TRH)	28	89	TSHB	81,567^b^	Thyroid stimulating hormone receptor (TSHR)	No

**Table 3:** Protein biomarkers for brain damage. Mass - the mass of the biomarker in kDa. Length – the length of peptide in amino acids

Marker	Definition	Alternative names	Mass (kDa)	Length (amino acids)	Coding gene	UniProt ID	Clinical trial
UFD1	Essential protein of the ubiquitin-dependent proteolytic pathway which degrades ubiquitin fusion proteins	Ubiquitin fusion degradation protein 1	34.5	307	UFD1	Q92890	NCT01833312
H-FABP	A protein that plays a role in the intracellular transport of long-chain fatty acids and their acyl-CoA esters, Lipid-binding, small cytoplasmic protein	Fatty acid-binding protein 3, Heart-type fatty acid-binding protein (H-FABP), Mammary-derived growth inhibitor (MDGI), Muscle fatty acid-binding protein (M-FABP)	4.858	133	FABP3	P05413	NCT01833312
IFABP	Intracellular protein, expressed abundantly in the intestine, involved in the intracellular transport of fatty acids; indicates intestinal damage	Fatty acid-binding protein 2, Intestinal-type fatty acid-binding protein, I-FABP	15.124	132	FABP2	P12104	No
PAI-1	Serine protease inhibitor	Endothelial plasminogen activator inhibitor, Serpin E1	45.06	402	SERPINE1 (PAI1, PLANH1)	P05121	NCT02310191 NCT03733223
S100B	Protein, calcium-binding, glial-specific	S-100 protein beta chain, S-100 protein subunit beta, S100 calcium-binding protein B	10.713	92	S100B	P04271	NCT00490295 NCT04795362 NCT04602325 NCT03919370 NCT03139682
CLU	Protein, disulfide-linked heterodimeric protein, member of the small heat shock protein family and, thus, a molecular chaperone	Aging-associated gene 4 protein, apolipoprotein J (Apo-J), complement cytolysis inhibitor (CLI), complement-associated protein SP-40, Ku70-binding protein 1, NA1/NA2, sulfated glycoprotein 2 (SGP-2), testosterone-repressed prostate message 2 (TRPM-2)	52.495	449	CLU (APOJ, CLI, KUB1)	P10909	NCT06034509
GFAP	GFAP, a class-III intermediate filament, is a cell-specific marker that, during the development of the central nervous system, distinguishes astrocytes from other glial cells	Glial fibrillary acidic protein	49.88	432	GFAP	P14136	NCT00756249 NCT03919370 NCT03176823 NCT02162654 NCT03139682
BDNF	Signaling molecule that activates signaling cascades downstream of NTRK2, neurotrophin family protein	Neurotrophin; abrineurin	27.818	247	BDNF	P23560	NCT02860260 NCT03903601 NCT01678534 NCT02856074 NCT01833312 NCT03139682
CRP	Acute-phase protein	C-reactive protein	25.039	224	CRP	P02741	NCT04475328 NCT01560520 NCT02484027 NCT02868723 NCT01446913 NCT00906542 NCT03868007
NR2A/2B	NMDA -receptor fragment, Glutamate [NMDA] receptor subunit epsilon-1 is a protein that in humans is encoded by the GRIN2A gene.	Glutamate [NMDA] receptor subunit epsilon-1, N-methyl D-aspartate receptor subtype 2 A (NMDAR2A, hNR2A), NR2A and NR2B-Containing NMDA Receptors; NR2A: NR2B subunit; NR2A/B; ; NMDAR2A; NR2A; glutamate [NMDA] receptor subunit epsilon-1; N-methyl D-aspartate receptor subtype 2 A; GRIN-2 A	165.283	1,464	GRIN2A	Q12879	No
D-dimer	Small protein fragment, fibrin degradation product	No	180 kDa	180–200	No	No	NCT01960478 NCT00206908 NCT03198715 NCT01833312
PCT	peptide precursor of the hormone calcitonin	Procalcitonin	12.640	116	CALC-1	No	NCT04247659 NCT01498705 NCT03899532 NCT03812666
SAA1	Major acute phase protein, produced by hepatocytes in response to infection, tissue injury and malignancy.	Amyloid protein A, amyloid fibril protein AA	12.500	104	SAA1	P0DJI8	NCT06327776
sNfL	Polypeptide filaments constituting a major component of the axonal cytoskeleton	Serum neurofilaments	No	No	No	No	NCT03139682

**Table 4:** Nucleic acid biomarkers for brain damage.^a^ - miRBase ID. ^b^ - RNA central ID

	Marker	Alternative names	Composition	Gene family	Coding number		Clinical trial
small coding RNA	miRNA-602	miR-602	23 nucleotides	MIPF0000767; mir-602	MIMAT0003270 ^a^		No
	miRNA-124	miR-124a/b	22 nucleotides	MIPF0000021; 8 chr (miR-124-1 and 2)	No		No
	rno-miRNA-122-5p	rno-miR-122-5p	22 nucleotides	MIPF0000095; mir-122	MIMAT0000827^a^		No
	rno-miRNA-300-3p	rno-miR-300-3p	22 nucleotides	MIPF0000018; mir-154	MIMAT0000902^a^		No
	miRNA-29b-3p	miR-29b-3p	23 nucleotides	MIPF0000009; mir-29	MIMAT0000100^a^		No
	miRNA-181a-5p	miR-181a-5p	23 nucleotides	MIPF0000007; mir-181	MIMAT0000256^a^		No
	miRNA-23b-3p	miR-23b-3p	23 nucleotides	MIPF0000027; mir-23	MIMAT0000418^a^		No
	miRNA-146b	miR-146b	73 nucleotides	MIPF0000103; mir-146	MI0003129^a^		No
	miRNA-221-3p	miR-221-3p	23 nucleotides	MIPF0000051; mir-221	MIMAT0000278^a^		No
	miRNA-382-5p	miR-382-5p	22 nucleotides	MIPF0000018; mir-15	MIMAT0000737^a^		No
	miRNA-let-7e-5p	miR-let-7e-5p	22 nucleotides	22q13.31 chr	No		No
	miRNA-21–5p	miR-21–5p	22 nucleotides	MIPF0000060; mir-21	MIMAT0000076^a^		No
	miRNA-494	miR-494	81 nucleotides	MIPF0000018; mir-154	MI0003134^a^		NCT03577093
long non-coding RNA	lnc-CRKL-2	No	1,666 nucleotides	lnc-CRKL-2, HSALNG0134182, NONHSAG033340.2 genes	URS00008BE552_9606 ^b^		No
	lnc-NTRK3-4	No	554 nucleotides	lnc-NTRK3-4 Gene	URS0000D6D860_9606 ^b^		No
	Inc-RPS6KA2-AS1	No	4,555 nucleotides	RNONHSAG045399.2, HSALNG0055114, RPS6KA2-AS1 genes	URS00008C23B8_9606 ^b^		No
	lnc-CALM1-7	No	4,841 nucleotides	ENSG00000275198, lnc-CALM1-7 genes	URS0000D5C183_9606 ^b^		No

## Results

### Enzymes

#### Nucleoside diphosphate kinase A (NDKA)

NDKA has been shown to be a brain-specific marker that is readily detected in blood samples. NDKA increases after the onset of a stroke [3]. This enzyme is encoded by the *NME1* gene and is involved in the exchange of phosphate groups between various nucleoside diphosphate groups [4]. It is expressed in neurons and involved in a cascade of reactions that occur during stroke, particularly during TIA [5, 6].

NDKA was identified as a biomarker for brain lesions and can be used for disorders such as head trauma, ischemic stroke (IS), hemorrhagic stroke, subarachnoid hemorrhage, and TIA [3, 4]. Based on the increase of NDKA within three hours of a stroke, this protein was proposed as a biomarker for the early diagnosis of stroke, with a sensitivity of 70–90% and a specificity of 90–97% [4, 7]. A subsequent study demonstrated that NDKA and PARK7 were significantly increased in stroke patients compared to controls (*p* < 0.0001), for NDKA the sensitivity and specificity were 90% [3, 4, 8].

#### Parkinson disease protein 7 (PARK7)

PARK7 is a protein found in many tissues, including the brain and heart. It is also referred to as an oncogene and is involved in cellular apoptosis [9, 10]. PARK7 has also been found in prostate tumors [11]. This enzyme is involved in the response to oxidative stress [12, 13] and is likely to be neuroprotective against oxidative stress and cell death [14]. PARK7 is an early plasma marker of stroke [3, 8]. After IS, PARK7 acts as an antioxidant in mitochondrial and neuronal nuclei. It downregulates pro-apoptotic factors such as p53 and inhibits apoptotic cell death [4, 15]. PARK7 is used as a biomarker for early diagnosis of stroke. It has been discovered to play a significant role in stroke due to the inflammatory processes that occur during an attack, which leads to the expression of α-synuclein and subsequent cell death. PARK7 protects against neuronal cell death by acting as a chaperone forming a complex with α-synuclein [3, 11, 14, 16]. After an ischemic event, PARK7 and NKDA are also elevated in the cerebrospinal fluid (CSF). PARK7 and NDKA are increased in various types of cerebrovascular injury [17, 18], and PARK7 may even play a role as a pharmacological target in the treatment of IS [19].

Ubiquitin fusion degradation protein-1 (UFD-1), along with PARK7 and NDKA, were first detected in post-mortem CSF samples of stroke patients, and subsequently confirmed as early plasma markers of stroke. Allard et al. compared stroke and TIA patients with healthy controls and were able to confirm elevated markers in three different cohorts [3, 8]. The study also showed that biomarker levels increased within three hours after stroke or TIA onset and remained elevated up to five days after onset.

#### Tissue-type plasminogen activator (tPA/PLAT) and plasminogen activator inhibitor 1 (PAI-1)

Tissue-type plasminogen activator (tPa) is an enzyme involved in the destruction of blood clots. It converts plasminogen to plasmin and serves as a marker for thrombotic/fibrinolytic activity [20]. tPA is used as a pharmacological agent in early treatment of stroke. Plasminogen activator inhibitor 1 (PAI-1) is a major tPA inhibitor [20]. One study has demonstrated diagnostic efficacy of tPA and PAI-1 in patients with suspected stroke or TIA [8]. However, tPA and PAI-1 were regarded to be unsuitable for use as single markers, but can add value in marker combinations [20].

PAI-1 along with tPA are markers of thrombotic/fibrinolytic mechanisms. tPA breaks down blood clots by catalyzing the conversion of plasminogen to plasmin and is used as a thrombolytic agent in the early stages of stroke. PAI-1 is a major tPA inhibitor. Both of these proteins were found to be elevated in a case-control study on patients with IS and the reported diagnostic value of these markers were very high (AUC tPA 0.97 and PAI-1 0.99) [21]. In patients with suspected stroke or TIA, both markers also showed diagnostic efficacy, albeit of limited value. tPA was an independent predictor of stroke in a study of 405 patients with suspected stroke [22]. tPA was also added to the hand-held facial speech test, and provided a modest but significant improvement in AUC. PAI-1 was assessed in addition to a clinical model in a sample of 278 patients with suspected stroke. In a model adjusted for age, gender, cardiovascular risk factors, and serum creatinine, PAI-1 remained an independent predictor of stroke [23]. However, PAI-1 did not remain an independent variable in the best diagnostic model, which consisted of age, face- arm- speech test (FAST), atrial fibrillation, and three other serum markers (protein S100B, MMP-9 and IL-6) [8]. According to a recent review, tPA and PAI-1 are not suitable as single markers, but may add diagnostic value when they are combined with other markers [20].

#### Matrix metalloproteinase-9 (MMP9)

Matrix metalloproteinase-9 (MMP-9) is a zinc-dependent extracellular peptidase that belongs to a subgroup of gelatinases. It is capable of modifying components of the extracellular matrix [24, 25]. MMP-9 is important for the normal functioning and development of the brain, but can be harmful in pathologies [25, 26]. MMP-9 acts on type IV collagen in the basement membrane of the endothelial walls, and elevated MMP-9 activity can lead to disruption of the blood-brain barrier (BBB), after cerebral ischemia and during inflammation. This allows leukocytes and cytokines to spread and enter the brain [25, 27].

During acute cerebral ischemia, α2-antiplasmin markedly enhances brain damage, disruption of the BBB, and increases the expression of MMP-9. α2-Antiplasmin is a plasmin inhibitor and impairs the dissolution of fibrin clots [28, 29]. α2-antiplasmin and MMP-9 have been shown to have similar deleterious effects on the ischemic brain [29]. Elevated blood α2-antiplasmin has been associated with an increased risk of IS in humans [28, 29]. High levels of α2-antiplasmin also causes disorders of the BBB, such as cerebral edema, microvascular thrombosis and brain cell death [29, 30].

#### Gamma-enolase (ENO2/NSE)

Gamma-enolase, or neuron-specific enolase (NSE) is an isomer of the intracytoplasmic lycolytic enzyme enolase, which is found in neuronal bodies, axons, as well as neuroendocrine cells, neuroendocrine tumors, and neuroectodermal cells [31, 32]. It has been investigated as an acute phase marker for stroke and may be a potential marker of early neuronal damage [31, 33]. Serum and CSF levels of NSE were found to peak 72 h after brain injury [31]. In hypertensive patients and patients with coronary artery disease, the average level of gamma-enolase in serum has been observed to be intermediate between IS and healthy controls. This may indicate that patients with diabetes have some degree of neuronal damage and/or damage to the BBB. This is supported by the observation of an increase in antibodies against gamma-enolase in patients with both type 1 and 2 diabetes [33].

NSE is also present in the saliva of stroke patients and patients at risk, which is due to disruption of integrity of the BBB and leakage of the enzyme outside the CNS [34, 35]. A positive correlation has been described between serum enolase (*r* = 0.56) and creatine kinase B-type (CKBB) (*r* = 0.53). These two markers are both described as sensitive and easy-to-follow markers for early diagnosis of cerebral ischemia. NSE can be used as a sensitive diagnostic or prognostic marker for diseases associated with cerebral ischemia [33].

NSE has also been used in TBI where higher concentrations of NSE have been found to be significantly associated with increased mortality and unfavorable outcomes [36].

#### Creatine kinase B-type (CKBB)

CKBB is present in neurons and astrocytes and penetrates from the cytoplasm into the extracellular fluid when cells are damaged. The CKBB content in CSF after stroke has been investigated, and peaks at approximately 48–72 h after stroke [31].

#### Sirtuin 2 (SIRT2)

Sirtuin 2 (SIRT2) is an NAD-dependent protein deacetylase that is most abundantly expressed in the brain [37, 38]. It is involved in the mediation of various processes associated with cerebral diseases, such as oxidative stress and neuronal inflammation [39]. Previous studies have shown that microglial SIRT2 expression is upregulated during stroke, which leads to inhibition of the anti-inflammatory function of infiltrating regulatory T cells [40]. SIRT2 and pro-inflammatory cytokines were examined and serum SIRT2 expression was found to be elevated in acute ischemic stroke (AIS) patients compared to controls, further analyses demonstrated that SIRT2 expression data could distinguish AIS patients from controls (AUC = 0.890, 78.7% sensitivity and 91.5% specificity). Serum SIRT2 levels were also positively correlated with NIH Stroke Score, tumor necrosis factor-α, IL-6 and IL-17. SIRT2 was suggested as a potential marker of risk and prognosis of AIS in clinical practice [41].

### Hormones and metabolites

#### B-type natriuretic peptide (BNP)

BNP has been suggested as a marker for stroke in a study that looked at the triage accuracy of a combination test of four blood markers (triage stroke panel, TSP). Its accuracy was compared to the MRI prehospital stroke scale (CPSS), which is commonly used to triage patients with suspected stroke. TSP uses BNP, degradation products of fibrin containing D-dimer, MMP-9 and protein S100B in whole blood or plasma samples, the results are presented as a single composite result, the Multimarker index (MMX). MMX values were found to be significantly higher in stroke patients compared with non-stroke patients (p 0.001). BNP values were also significantly higher in stroke patients. However, 25% of patients with a definitive stroke diagnosis were not identified on triage using the TSP analysis. The combination of TSP and CPSS results may however improve accuracy over either test by itself [42].

#### Thyroid hormones: triiodothyronine (T3), thyroxine (T4), thyroglobulin (tg) and thyroid-stimulating hormone (TSH)

Thyroid hormones have been investigated as markers of acute systemic hypoxia/ischemia post-mortem to detect deaths caused by neck compression asphyxiation. These data demonstrate that elevated levels of thyroid-related hormones (T3 and T4) are indicative of systemic hypoxia/ischemia and brain damage due to several factors: mechanical asphyxia, acute/subacute blunt head trauma, acute/subacute blunt non-head injury trauma with a sharp instrument as a state of hemorrhagic shock, drowning, burning, and death due to cardiac dysfunction. Blood samples were taken from the left and right chambers of the heart and iliac veins and hormones levels were measured by electrochemiluminescence immunoassay. Serum T3 levels were higher with injuries such as mechanical asphyxia and acute/subacute blunt head trauma and were indicative of hypoxic and ischemic brain conditions [43]. In the same study a strong correlation was present between T3 and T4 levels at all blood sampling sites. Similarly, T4 levels were higher in asphyxia samples compared to deaths from other causes other than acute/subacute blunt head trauma-samples, regardless of where the samples were collected (*p* < 0.05–0.001) [43].

Elevated Tg levels were observed in cases of asphyxia compared to cases of fire fatality, in blood samples from the right cardiac chamber and iliac vein, and the left cardiac chamber in cases of fire fatality and drowning (*p* < 0.05). In addition to post mortem data, T4, T3 and Tg levels were elevated after exposure of thyroid cell lines to hypoxia for 10–30 min [43]. These data indicate that systemic hypoxia/ischemia can induce rapid release of thyroid hormones T3 and T4. The results of the study demonstrate that thyroid hormones have potential use as indicators of systemic hypoxia/ischemia postmortem [43].

Eighteen metabolites were identified to be associated with AIS in a study on 99 patients: oleic acid, linoleic acid, cer (d18:0/14:0), cer (d18:0/16:0), arachidonic acid, non-adecanoic acid, docosahexaenoic acid 4-hydroxyproline, pe[18:2(9z,12z)/18:1(9z)], pe[18:2(9z,12z)/18:0], l-palmitoylcarnitine, propionylcarnitine, tetradecanoylcarnitine, l-glutamine, l-arginine, dodecanoylcarnitine, l-proline and decanoylcarnitine [44]. These represented metabolic pathways such as fatty acid- and amino acid metabolism. Furthermore, this study identified differences in the metabolic profile between patients with large artery atherosclerosis and small artery occlusion. The results show that these metabolites can be used as markers for distinguishing between different subtypes of IS.

### Other proteins

#### Heart-type fatty acid binding protein (H-FABP)

Heart-type fatty acid binding protein (H-FABP) is a small cytoplasmic protein that plays a role in the intracellular transport of long-chain fatty acids and their acyl-CoA esters [45]. This protein was initially detected in the myocardium, but is also contained in CNS neuronal cell bodies and is rapidly released after ischemic damage to neurons. H-FABP is also a marker of stroke [46] and several studies have demonstrated positive correlations between IS and H-FABP levels [23, 45, 47, 48]. Both H-FABP ELISA kits and on-site tests are available.

A pilot study that included 22 cases (eleven IS, six intracerebral hemorrhage (ICH), five TIA) and 22 controls demonstrated that H-FABP had a sensitivity of 68.8% and specificity of 100% in the studied conditions [3]. However, a study with a larger population (111 IS and 127 controls with other neurological diagnoses) found lower accuracy: sensitivity was 59.5% and specificity was 79.5% [48]. Based on these data, H-FABP has been suggested to not be suitable for use as a single marker due to its limited sensitivity for ischemic insult, but may have diagnostic value when it is combined with other markers [20].

#### Protein S100-B (S100B)

S100B is a calcium-binding protein that is present in high concentrations in glial cells and Schwann cells. During cerebral ischemia, S100B was found to be abundantly expressed in microglia. Microglia function as an immunological defense against brain injury and quickly migrate to the site of injury during cerebral ischemia to initialize the release of effector molecules and the recruitment of other immunoreactive cells [49, 50]. S100B is a member of the Ca2^+^-binding protein family and is involved in the regulation of both intracellular and extracellular activity in the nervous system [51, 52]. S100B levels are predictive of complications after stroke-induced ischemic injury [53, 54]. S100B expressed during cerebral ischemia, is capable of causing neuronal damage by inducing overexpression of nitric oxide synthase and subsequent release of nitric oxide [55], activation of NF-κB in response to inflammation [14], and activation of microglia [56].

Microglial S100B expression has also been found to increase with time, starting 1–3 days after middle cerebral artery occlusion in a mouse model for stroke (MCAO), and reaching a peak after five days and then persisting up to 14 days. Experiments showed that increases in S100B expression were gradual between six and twelve hours after treatment with M1 stimuli (LPS plus IFNγ) and persisted for at least 24 h [57]. S100B pretreated microglia also showed increased migratory activity.

Interestingly, S100B was found to aggravate cerebral ischemia in MCAO mice by stimulating the polarization of the M1 microglia, stimulating MCP-1-induced microglial migration towards injured or inflamed brain tissue, induce expression of CCR2 and antagonizing the protective properties of M2 microglia [57]. In a study from the USA: NSE, S100B and CKBB in CSF were investigated as predictors of outcome in stroke patients. S100B was found to have the highest expression during the first 24 h after oxygen deprivation injury and then decrease over the next 48 h [31]. Interestingly, the expression of S100B is regulated by miR-602 [58], changes in expression of both miR-602 and S100B in have been observed in stroke patients [59]. S100B and miR-602 can both be considered suitable diagnostic markers [58].

Several studies have demonstrated that serum S100B levels may be negatively correlated with IS and higher serum S100B levels may contribute to a worsening condition and prognosis for IS patients [60]. S100B is also included in the Triage® Stroke Panel, which is a rapid fluorescence immunoassay for quantitative measurement of BNP, fibrin degradation products containing D-dimer, MMP-9 and S100B in whole samples blood or plasma. The MMX value obtained by combining the results of four different markers (MMP-9, BNP, S100B, and D-dimer) was significantly higher in stroke patients compared to non-stroke patients [42]. S100B has been extensively studied in TBI and has been suggest as a diagnostic biomarker of mild TBI as well as predict secondary insults in severe TBI has been suggested to contribute to diagnostics in combination with glial fibrillary acidic protein (GFAP), UCH-L1, NfH, NfL, microtubule-associated protein tau (tau), H-FABP, SNTF, NSE, microRNAs (miRNAs) and myelin basic protein (MBP) [61, 62].

Another recent systematic review and meta-analysis compared the diagnostic and prognostic accuracy of S100B and NSE for TBI. These biomarkers have demonstrated value in predicting outcomes, especially in regard to mortality, but were not useful for assessing abnormal CT results [63]. The S100B biomarker, when used in routine practice, has shown only limited clinical value in the diagnosis of mild TBI and in elderly patients. The authors suggest this to be due to low specificity. The researchers believe that combining several biomarkers may be useful for improving the accuracy of mild TBI detection [64]. To date, S100B is the most widely studied biomarker, and is also included in some clinical recommendations of TBI diagnostics [65, 66].

#### Glial fibrillary acidic protein (GFAP)

GFAP is a cell-specific marker that is found in astrocytes during the development of the CNS. During CNS damage, GFAP is cleaved by caspases and GFAP levels in the blood have been found to reflect processes associated with various types of CNS damage. A previous study determined the neoepitope that is generated by caspase-6-mediated cleavage of GFAP (GFAP-C6) and assessed the ability of GFAP-C6 to reflect pathological processes in patients suffering from cardiac arrest (CA) and subsequent global cerebral ischemia [67]. The results showed that GFAP-C6 levels did not correlate with other markers of CNS damage.

In TBI, cerebral ischemia, as well as neurodegenerative diseases, astrocytes are activated [68–70]. The activation of GFAP, which is the main component of intermediate filaments in astrocytes, occurs in parallel with this process [69]. GFAP and its unspecified degradation products (GFAP-BDP) have been considered as possible markers for various types of CNS damage [71–74]. Several studies have shown that GFAP levels are elevated in CSF and blood in patients with mild to severe TBI, and GFAP levels to reflect the severity of injury [71, 72]. Furthermore, it has been shown that serum GFAP, measured within 24 h after injury, outperforms clinical characteristics in predicting CT abnormalities [75]. An equally interesting fact is that GFAP levels in the CSF make it possible to distinguish between patients with IS and healthy people within the first 24 h after trauma, and that GFAP correlates with the severity of stroke [73, 74]. The authors believe that changes in GFAP levels reflect processes associated with different types of CNS damage. The degree of detail on processes underlying CNS injury, provided by a biomarker, might increase by targeting disease-specific posttranslational modifications (PTM) of proteins as biomarkers. Fragmentation of proteins by caspases is one possible process resulting in PTMs [76, 77]. Pro-caspase-6 is observed in astrocytes after transient focal cerebral ischemia in rats, and active caspase-6 is localized together with GFAP in the brain of patients with HIV-associated dementia [78, 79]. Authors hypothesize that GFAP-C6 may reflect the pathological processes underlying neurodegeneration as a function of cerebral ischemia resulting from CA.

Several publications on this subject suggest that GFAP levels increase 24–48 h after CA, showing differences between groups of results twelve hours after injury [80–82]. This is consistent with a hypothetical model of the temporal profile of markers of post-CA brain damage, in which GFAP is classified as a “persistent acute marker” that increases approximately twelve hours after CA, peaks at 24 h, and reaches pre-CA levels between 48 and 96 h [83]. Within the available time frame of this study, serum GFAP-C6 levels did not indicate neurological outcome 180 days after CA. However, since increases in serum GFAP-C6 levels do not begin until 72 h after injury, it is possible that GFAP-C6 levels do reflect pathological processes in the damaged CNS, and thus levels at later time points may indicate neurological disorders [67]. GFAP-C6 did not correlate with confirmed fluid biomarkers of brain damage such as NSE, S100B or tau at any time point.

GFAP levels in serum were also considered as possible biomarkers of differentiation between AIS and ICH. In a meta-analysis where data from 340 patients from four studies were collected, serum GFAP was found to be elevated in participants with ICH compared with AIS. No association was found with disease outcomes [84].

#### Brain-derived neurotrophic factor (BDNF)

Brain neurotrophic factor (BDNF) is a protein in the neurotrophin family that plays a vital role in neuronal development, regeneration, and plasticity [85]. Reduced BDNF can be an indicator of AIS [86]. A study by Wang et al. showed that serum BDNF levels are lower in AIS patients compared to healthy people [86]. BDNF and miRNA play an important role in the pathogenesis of AIS. A study from Tong Ren Hospital, Shanghai, China researched BDNF and miR-124 expression in AIS patients [86]. Serum samples were examined from AIS patients and healthy subjects to determine the levels of BDNF and regulatory miRNAs involved in AIS. Serum BDNF levels were measured in AIS patients and in healthy controls. This study also tested the effects of miR-124 on BDNF expression, which was studied in human neuronal cell lines. The results show that BDNF levels were reduced in the AIS patients while miR-124 levels were increased.

BDNF and BDNF-regulatory miR-124 have been suggested to be molecular markers of AIS. National Institutes of Health Stroke Scale (NIHSS) scores were negatively correlated with BDNF levels in a previous study on AIS patients, but positively correlated with miR-124 [86].

Lower serum BDNF levels were observed in the unfavorable group compared to the favorable group, likely due to increased autonomic function and BBB disruption. Conversely, higher CSF BDNF levels were found in the unfavorable group, likely attributed to increased BBB breakdown and enhanced transfer of serum BDNF to the brain [87].

#### NR2A, NR2B fragments of NMDA receptor (GRIN2A)

Peptide fragments of NR2A and NR2B car occur as a result of cleavage of synaptic receptors of N-methyl-D-aspartate. The excitatory receptor N-methyl-D-aspartate (NMDA) is one of the key regulators of the cerebral ischemic cascade. NR2A/2B fragments can cross the BBB and enter the bloodstream immediately after an episode of cerebral ischemia [20].

#### Markers of intestinal mucosa integrity

The intestinal mucosa integrity markers D-lactate and Intestinal Fatty Acid-Binding Protein (IFABP) are of particular interest for use as markers of brain ischemic lesions as these have been observed to be elevated within 24 hours of an AIS [88]. After IS, patients may experience complications that are unrelated to the CNS, such as pneumonia, urinary tract infections and deep vein thrombosis. In addition, gastrointestinal complications are also quite common, but the mechanisms behind these phenomena are as of yet unresolved. Work on animal models have shown that a disruption of the intestinal barrier can occur after an IS. However, there was no correlation between D-lactate and IFABP levels and the occurrence of an inflammatory response. Also, D-lactate and IFABP levels were not associated with patient’s outcomes.

#### Cerebral vascular markers

An IS can result from clotting in the arteries, which decreases cerebral blood flow and causes ischemic damage with subsequent destruction of the BBB, cerebral edema, hemorrhage, and cell death [89]. α2-antiplasmin markedly enhances brain damage, disruption of the BBB and MMP-9 expression. Both α2-antiplasmin and MMP-9 are released in the damaged brain and have similar deleterious effects. PAI-1 and tPA are markers of thrombotic and fibrinolytic mechanisms, respectively, and also have potential as markers of cerebrovascular brain damage.

#### Inflammatory markers

C-reactive protein (CRP) is a protein of the acute phase of inflammation, the concentration of which increases thousands of times at sites of infection or inflammation. CRP is synthesized mainly in liver hepatocytes, but also in smooth muscle cells, macrophages, endothelial cells, lymphocytes and adipocytes [90]. CRP is considered to be a non-specific marker of inflammation that is produced downstream of IL-6 signalling. A previous study assessed the role of CRP, leukocyte count, and d-dimer as predictors of delayed cerebral ischemia (DCI) [91]. Patients who were admitted within 24 h of aneurysmal subarachnoid hemorrhage (aSAH) were included in the study. CRP, d-dimer, leukocyte count and procalcitonin were assessed at admission and on days one, four, nine, 14 and at discharge. The results of the study showed that serum CRP levels were higher in patients with severe vasospasm. The results of the study confirm the multifactorial genesis of DCI, including vasospasm, microthrombotic and inflammatory processes. The aim of the study was to identify easy-to-use markers for patients at increased risk of DCI, vasospasm, and poor outcome after aSAH. Leukocyte counts and CRP had no predictive value in patients examined within 24 h after inner cerebral trauma (ICT). However, CRP levels were higher at the critical time in patients with angiographic confirmation of vasospasm. The authors suggest that enhanced multimodal monitoring and strict imaging control be observed in this patient population.

Endocan, an endothelial inflammatory marker that is associated with cardiovascular disease, has recently been proposed as a marker of silent brain infarction (SBI), which is considered to be a subclinical risk factor for future stroke. In a study including 54 SBI patients and 52 controls, serum levels of endocan and high sensitivity CRP were found to be higher in patients with SBI compared to controls [92].

The role of the serum marker d-dimer as a prognostic factor for the onset of DCI has previously been evaluated [91]. Authors analyzed all patients admitted within 24 h after aSAH. The Fisher and Glasgow Scales were used to assess the condition. Results show that DCI correlates with severe vasospasm and poorer outcome. D-dimer levels on admission were correlated with Fisher scores. DCI was more likely to occur in patients with Fisher grade IV hemorrhage if d-dimer levels were higher on admission. The authors suggest that serum d-dimer levels above 0.445 mg/ml may be a predictor of DCI in patients with Fisher grade IV aSAH.

The other investigated markers, leukocyte count and CRP, had no predictive value in patients examined within 24 h of ICT. Elevated d-dimer levels in patients with Fischer grade IV aSAH on admission were correlated with an increased risk of DCI. In the late phase (days 9 and 14), d-dimer levels increased with the onset of DCI [91]. D-dimer has also been proposed as an independent marker of AIS caused by large vessel occlusion [93].

Procalcitonin (PCT) is a peptide precursor of the hormone calcitonin. It is produced by C-cells of the thyroid gland. Preprocalcitonin is cleaved by endopeptidase. PCT is a marker of infection [94], but recent studies have demonstrated its potential as an IS marker [95]. PCT was identified retrospectively in 748 patients’ serum less than 72 h after symptoms of brain damage. Inflammatory markers including PCT, CRP and neutrophil levels were increased in patients with adverse functional outcomes than patients without such outcomes (1.37 ± 1.81 ng/mL vs. 0.12 ± 0.41 ng/mL). In addition, PCT, CRP, and neutrophil percentage, were associated with 30-day mortality. Results of the study suggests that inflammation may accompany stroke, and PCT may play a role as prognostic marker of 30-day mortality [96].

Serum Amyloid A1 (SAA1) is an acute phase protein that is primarily produced by hepatocytes in response to infection, tissue damage and malignancy [97]. SAA1 is the main precursor of amyloid A, the deposition of which leads to inflammatory amyloidosis [98, 99]. In a recent study, SAA1 was used as a biomarker for TBI. Patients with TBI may experience intracranial and extracranial injuries. Extracranial injury is a more common complication in TBI patients and can lead to worse outcomes. Also, hypotension and hypoxemia can accompany extracranial damage, which complicates the course of the disease due to the difficulty of their identification and detection. In the study, SAA1 was associated with the volume of traumatic intracranial lesions. SAA1 levels also correlated with markers such as S100B and GFAP, NSE, tau and phosphorylated axonal neurofilament subunit H (pNFH). SAA1 was associated with poor outcome and mortality at hospital discharge and after six months. The authors suggested that SAA1 can be used as a marker of the patient’s general condition due to its involvement in the neuroendocrine systemic response to TBI [100].

#### Markers of neuronal damage

Clusterin is a cytoprotective chaperone that is released by neurons in response to neurological damage. It is constitutively expressed in a wide variety of tissues [101] but also in neuronal tissue by subpopulations of neocortical neurons, hippocampal/neocortex glial cells, and ependymal cells [102, 103]. This protein is involved in a variety of cellular processes, including apoptosis, cell cycle regulation and DNA repair [104]. High levels of clusterin immediately after ischemic injury have been demonstrated in vivo [105].

Clusterin binds to a wide range of misfolded proteins to inhibit the formation and accumulation of toxic protein complexes, e.g. amyloid complexes [106, 107]. Previous studies also point towards a protective effect of clusterin against Alzheimer’s disease [108]. In ischemia studies, clusterin has been found to initially be decreased after twelve hours, and then increased after 48 h [109]. Clusterin measured in CSF and plasma has been suggested as a biomarker for neurological injury.

Serum neurofilaments light chain (NfL) is one of the subunits of neurofilaments, along with the middle chain (NfM), heavy chain (NfH) and alpha-internexin [110]. Serum neurofilaments (sNF) themselves are 10 nm polypeptide filaments that constitute a major component of the axonal cytoskeleton. After neuroaxonal injury, neurofilaments are released into the extracellular space, CSF, and peripheral blood [110]. Serum NfL can indicate neuro-axonal damage [111]. The detection of neurofilament light chain samples in peripheral blood has only become possible in recent years [112]. However, in AIS and TIA, the role of serum NfL has not yet been explored in large study groups. A recent study tested the association of serum NfL with clinical severity at admission, diagnosis of AIS versus TIA, infarct size at admission and functional outcome after three months. Patients with AIS or TIA had higher serum NfL levels compared to healthy volunteers. Clinical severity at admission was associated with NfL levels obtained in the first 24 h after the onset of stroke. Findings also support the concept that serum NfL reflects damage to axons. Among AIS patients, infarct size was not significantly associated with serum NfL levels at the time hospital admission. From the day of admission to the next, patients with extensive infarctions showed significant increases in NfL levels, which suggests that these only peak after a few days. The three-month outcome after adjusting for established predictors was not associated with NfL levels at admission.

Two previous studies compared AIS patients with healthy controls and found higher blood NfL levels among AIS patients [113, 114]. Among patients with AIS, clinical severity on admission was associated with NfL in the blood [113]. A study involving 18 patients with AIS did not find a significant association between NIHSS-scores and sNf [114]. In one study, serum NfL in TIA-patients was 1.7 fold lower than in patients with AIS, while a study of patients with nontraumatic cervical artery dissection showed that NfL was 6.6 fold lower than in patients with AIS. Here, NfL was assessed within 30 days of the onset of symptoms [115]. Over a longer time-frame, more NfL is released from damaged axons into the bloodstream, a phenomenon that may explain the large difference between TIAs in AIS observed when assessing NfL in blood taken within 30 days of symptom onset. Serum NfL is also a marker of neurological disorders: multiple sclerosis, frontotemporal dementia, amyotrophic lateral sclerosis, and some acute neurological disorders such as acute spinal injury and cranial brain injury [116–118].

Serum NfL shows promise as a biomarker for both acute and repetitive sports-related concussions, as well as for individuals with subacute and chronic TBI. It serves as a sensitive and clinically significant indicator of axonal injury caused by TBI, accurately reflecting the extent of underlying damage. This validation is supported by advanced MRI, cerebral microdialysis, and an experimental model [119, 120].

NfL may play a role as a novel biomarker in patients with MELAS (mitochondrial encephalomyopathy lactic acidosis and stroke-like episodes), a progressive neurodegenerative disease. NfL is also accompanied by symptoms such as diabetes, seizures, hearing loss, heart disease, short stature, endocrinopathies etc [121, 122]. This was found in a study that included 23 patients with MELAS, 15 in the acute phase of MELAS and ten in remission. Serum NfL levels increased significantly in MELAS patients. Serum NfL levels in the acute attack group (146.73 [120.91-411.31] pg/ml, median [IQR]) were higher than in the remission group (40.31 [19.54-151.05 ] pg/ml, median [IQR]) and the healthy control group. (7.70 [6.13–9.78] pg/ml, median [IQR]). The NfL level in the remission phase group was higher than in the control group (*p* < 0.05) and correlated with the lesion volume in the brain (*r* = 0.740, *p* < 0.001).

Recent studies have focused on the use of biomarkers as a diagnostic method for TBI [63, 123–125]. GFAP, S100B and NfL have shown to be promising biomarkers in the diagnostics of TBI, but large-scale systematic studies are needed to study markers in different clinical groups [126].

TBI is associated with a broad array of pathological conditions: from mild changes in consciousness to inexorable coma, brain ischemia and death. Markers that are elevated during these conditions due to TBI are of great importance. Previously we mentioned PARK7 and NDKA as indicators of various types of cerebrovascular injury. *Galectins* are another class of proteins that are expressed in tissues relevant to cerebrovascular accident and have been suggested as suitable biomarkers [127].

### Nucleic acids

#### Micro-RNAs as potential biomarkers

MicroRNAs were first discovered in the early 1990s and have been considered as markers of pathological conditions such as cardiovascular disease and different types of cancer [128–130]. MiRNAs can also be used as markers for pathological conditions of the CNS. MicroRNAs are involved in the regulation of cellular behavior and various metabolic pathways. Several studies have examined the principles of regulation of miRNA signaling pathways of stress and applied these concepts to understand the role of miRNAs in disease [131].

MiRNAs are small regulatory RNA molecules that integrate into RNA-induced silencing complexes, which bind to partially complementary sequences in the target mRNA and modulate gene regulation by inhibiting translation and destabilizing transcripts. Each mature miRNA may have the ability to regulate the expression of many genes [132].

Thanks to technological developments, detection methods for quantification of miRNAs have become readily available. Expression of many different miRNAs in parallel can be measured using deep sequencing and microarray analysis, while the level of individual miRNAs can be determined using Northern blotting, real-time PCR and in situ hybridization [133]. Currently, original studies are underway in both humans and animals to identify miRNAs and their role in the regulation of the synthesis of specific proteins [55, 132, 134].

MiRNAs have generated great interest as potential markers for the early stages of brain damage and have the potential to be clinically beneficial. MiRNAs are noncoding, highly conserved RNAs approximately 18–25 nucleotides in length that suppress their target mRNAs by modulating post-transcriptional gene expression [135]. miRNAs are further processed to mature miRNAs, where they partially and specifically bind to the 3’-untranslated region of the target mRNA and thus suppress translation [135, 136]. miRNAs are involved in various pathophysiological processes [131, 133] and can play a role as potential non-invasive biomarkers for various diseases, since they are stably found in biological fluids [128–130]. Studies have shown that certain miRNAs are expressed in the CNS and may be involved in the pathogenesis and progression of neurological disease [137, 138]. Therefore, miRNAs have also been hypothesized to be involved in the molecular mechanisms underlying hypoxia/ischemia-mediated dysfunction and neuronal damage [139]. MiR-9 and miR-124a are highly expressed and associated with AIS [140]. Upregulation of miR-147 and miR-34a has also been observed to modulate inflammation [132]. Recently, circulating miRNAs have been introduced as sensitive biomarkers of brain damage [46, 141, 142]. These can be useful biomarkers for the diagnosis of IS and for monitoring disease outcomes [58]. mRNAs are also gaining increased interest as biomarkers for Alzheimer’s disease [143].

#### Small coding RNAs

MiR-602 expression and its potential target, S100B, have been assessed as potential diagnostic and prognostic markers of IS [58]. Studies have previously demonstrated altered expression of miR-602 and S100B in patients with stroke [59]. In addition, the expression of BDNF and miR-124 in have also been measured in AIS patients. The effect of miR-124 on BDNF expression has also been investigated in human neuronal cell lines. Results showed that BDNF levels were reduced in AIS patients and that BDNF was negatively correlated with miR-124 levels. BDNF was also positively correlated with the severity of stroke as assessed by the NIHSS. A positive correlation was observed between NIHSS-scores and miR-124 expression. The results of the study point to the possible use of serum BDNF and miR-124 as molecular markers for AIS [86].

Another recent study examined the expression patterns of specific miRNAs in patients with TIA. 754 miRNAs were initially tested using the TaqMan Low Density Array (TLDA) in two pooled serum samples from 50 patients and 50 controls. The most significant miRNAs were subsequently investigated using individual quantitative reverse transcription PCR (qRT-PCR) assays, first in the same TLDA cohort, and then confirmed in another larger cohort, including 177 IS patients, 81 TIA patients, and 42 controls. TLDA screening showed that 71 miRNAs were activated and 49 miRNAs were suppressed in IS patients. qRT-PCR verification confirmed the serum levels of miR-23b-3p, miR-29b-3p, miR-181a-5p, and miR-21-5p to be increased in patients with IS. Interestingly, serum levels of miR-23b-3p, miR-29b-3p, and miR-181a-5p were also increased in TIA patients. The results demonstrate that these miRNAs can function as prognostic biomarkers for IS and TIA, and their distinctive expression signatures can help assess the severity of IS neurological deficit and the subsequent risk of stroke after TIA [139].

An additional study measured 17 different mRNAs (Let-7b, miR-23a, miR-126 miR-15a, miR-16, miR-17-5p, miR-19b, miR-29b, miR-339-5p miR-21 miR-221 miR-32-3p, miR-106-5p, miR-532-5p, miR-146b, miR-210, miR-145) and tested these for association with stroke in 128 patients with AIS. An increase in miR-146b in serum was found within 24 h after the onset of stroke. In addition, up-regulation of serum miR-146b was strongly correlated with plasma CRP, infarction volume, and NIHSS scores, and also correlated with serum IL-6. Importantly, the combination of plasma hs-CRP and serum miR-146b provided the best sensitivity/specificity for predicting AIS (AUC 0.782 to 0.863). Authors suggested that upregulation of serum miR-146b in AIS may be a potential biomarker for assessing AIS [144].

In a study by Huang et al. the miRNA let-7e-5p was associated with IS. Results showed that serum levels of let-7e-5p were elevated in patients with IS compared to controls. The addition of let-7e-5p to traditional risk factors resulted in an improvement in prediction models as AUC increased from 0.74 (95% CI, 0.70 ∼ 0.78) to 0.82 (95% CI, 0.78 ∼ 0.85). Cellular experiments showed that expression levels of four genes enriched in the MAPK signaling pathway were inhibited by let-7e-5p transfection. In particular, the levels of expression of the *CASP3* and *NLK* were reduced in patients with IS and were also negatively correlated with let-7e-5p expression. These studies indicate the use of let-7e-5p as a potential biomarker of IS and indicate its involvement in the pathological mechanisms of IS [145].

The effect of miR-494 on histone deacetylase 2 (HDAC2) -mediated neutrophil infiltration and phenotypic shift was assessed in a recent study. MiR-494 levels in neutrophils from AIS patients were assessed by real-time PCR and found to be increased. Chromatin immunoprecipitation found that HDAC2 targeted genes for multiple matrix metalloproteinases and Fc-gamma receptor III (CD16) in neutrophils. A chemically engineered antagonist for mir-494, antagomiR-494, was found to suppress the expression of several MMP genes, including MMP7, MMP10, MMP13, and MMP16, and decrease the number of neutrophils that were able to invade the brain in mice [146]. AntagomiR-494 also inhibited neutrophil displacement to the pro-inflammatory N1 phenotype in vivo and *in vitro.* The authors suggested that miR-494 may serve as a predictive biomarker of disease outcome after stroke.

In a study on rats, serum and CSF-levels of miR-122-5p and miR-300-3p were assessed as TIA markers after focal cerebral ischemia caused by middle cerebral artery occlusion (MCAO). Plasma miR-122-5p was suppressed in rats after ten minutes of ischemia and miR-300-3p was increased. ROC analysis showed high AUC values for miR-122-5p (0.960) and miR-300-3p (0.970). MiR-122-5p and rno-miR-300-3p may be suitable as blood-based TIA biomarkers [134].

#### Long non-coding RNAs

Long non-coding RNAs (lncRNAs) have also been investigated as markers of ischemic brain damage. One study investigated the relationship between lncRNA expression and acute minor stroke (AMS), which is difficult to diagnose due to the lack of effective molecular markers. AMS is a type of hypoxic ischemic necrosis with no more than four points on the NIHSS. Recently, many lncRNAs associated with AMS have been gradually discovered. Using RNA-seq, lncRNAs were identified in exosomes isolated from the serum of AMS patients. Expression levels were then assessed using RT-PCR. Results showed that lnc-CRKL-2 and lnc-NTRK3-4 were increased in AMS patients, while the expression levels of RPS6KA2-AS and lnc-CALM1-7 were sharply reduced. The authors believe that these four lncRNAs can be used as biomarkers for the early detection of AMS [147].

### Perspective

Through literature searches and a review of the clinicaltrials.gov database, we were able to identify 41 different biomolecules that are either already used in indicators of ischemia in clinical diagnosis, or have been proposed as potential biomarkers of brain damage. Several of the potential biomarkers are also supported by results from clinical and preclinical research.

#### Acute and early stage diagnostic markers

Biomarkers play a crucial role in assessing various stages of brain damage, but their utility can be influenced by several factors, including sample source, timing of measurement, half-life, and the choice of analysis method. The biomarkers can be indicators of the different specific stages of brain damage: this includes biomarkers as acute, early and late stage phase markers, as well as markers for monitoring. For example pro-inflammatory cytokine IL-17A levels rise within 12 h after reperfusion in CSF and in serum and peak on the third day of perfusion after an IS. NDKA and PARK7 can be considered to be acute stage markers, as they can be detected within three hours after stroke [7]. CRP is another marker that can be used as a marker of the acute phase. Interestingly, serum and CSF levels of miRNA-let-7e-5p have been associated with AIS and patients displayed elevated levels of miRNA-let-7e-5p at the acute stage [145]. According to the available data, CKBB levels are elevated during the first 24 h after anoxic injury and then declines over the next 48 h [31]. Research also demonstrated serum NSE to increase after anoxic injury and peak after 72 h [31].

It can be a challenge to determine the optimal time for biomarker assessment. The release and concentration of biomarkers may vary depending on the specific stage of brain damage, which can make it challenging to capture the most accurate and representative measurement. Additionally, the half-life of biomarkers can affect their usefulness. Some biomarkers may have a short half-life, meaning they are quickly cleared from the body, while others may have a longer half-life, allowing them to persist in measurable levels for a more extended period. Understanding the half-life of a biomarker is important for determining the appropriate timing of measurements and interpreting the results accurately.

Moreover, the choice of sample collection source is critical for biomarker analysis. For instance, biomarkers can be measured in blood, CSF, or other bodily fluids such as saliva. Each sample type has its own advantages and limitations, and the choice depends on factors such as invasiveness, accessibility, and correlation with the specific brain injury being assessed. Furthermore, the selection of an appropriate analysis method is crucial to accurately interpret biomarker measurements. Various techniques, such as immunoassays, mass spectrometry, and molecular diagnostics, are employed to analyze biomarkers. Each method has its own sensitivity, specificity, and limitations, and careful consideration must be given to selecting the most suitable analysis method for the biomarker of interest. To maximize the utility of biomarkers, researchers and clinicians must consider these factors and carefully select the appropriate timing, sample collection method, and analysis techniques. This comprehensive approach ensures that biomarker measurements provide meaningful and reliable insights into the stages and progression of brain damage [148–150].

#### The benefit of multiplexing

A systematic review from 2021 found that a combination of serum markers may have a higher diagnostic value than each marker alone. The authors proposed three panels to be evaluated in future studies. Panel 1: NR2, GFAP, MMP-9, von Willebrand factor (vWF) and S100B; Panel 2: NR2, GFAP, MMP-9, vWF and the ischemia-modified albumin index (IMA); and Panel 3: NR2, GFAP, antithrombin III (AT-III) and fibrinogen [151]. NR2 and GFAP are brain-specific markers associated with stroke. vWF, MMP-9 and S100B are widely used as biomarkers, while others such as IMA, AT-III and fibrinogen have not been evaluated in combination.

A combination of ten serological markers was also recently proposed for assessing the risk of acute cerebral infarction [152]. This included total cholesterol, triglycerides, high-density lipoprotein cholesterol, low density lipoprotein cholesterol, high sensitivity cholesterol, CRP, homocysteine, lipoprotein-linked phospholipase A2, ischemic-modified albumin, complement C1q and lipoprotein. An analysis in 154 patients with acute ischemic cerebral infarction found that individual markers had a lower predictive value in the diagnosis of acute cerebral infarction compared to a combination of all nine markers, and the authors proposed that a combination of six to seven markers should significantly increase the potential for early diagnosis [152]. In another recent study, a group of scientists from China analyzed 13 metabolic markers in serum (5-aminoimidazole-4-carboxamide, dimethyltryptamine, 1-hexadecanoyl-sn-glycero-3-phosphoethanolamine, palmitic acid, pipecolic acid, 1-heptadecanoyl-sn-glycero-3-phosphocholine, linoleic acid, tyramine, vanylglycol, l-2,4-diaminobutyric acid, 2-phenylglycine, 5,6-epoxy-8,11,14-eicosatrienoic acid, and l-tyrosine) and found that a combination of ten metabolites had predictive value for diagnosis of cerebral infarction (AUC > 0.7) [153].

Recently, neuron-specific proteins were linked to infarct volume through analyses of eight brain-enriched candidate proteins (GFAP, MBP, β-synuclein, OPALIN, MT-3, SNAP-25, KIF5A, MOBP) [154]. This study included 43 patients with IS and blood was drawn immediately after hospital admission. A positive correlation was observed between serum levels of all eight proteins with infarct volume. It is also worth noting that the most significant correlation was observed for four neuron-specific proteins (MT-3, SNAP-25, KIF5A, β-synuclein). The strongest correlation with infarct volume was given by a combination of these four proteins [154].

#### Neuroimaging in comparison with biomarkers

There is still a strong reliance on CT and MRI as leading diagnostic methods in identifying various brain pathologies, including brain damage, ischemic stroke, and traumatic brain injury. CT scans provide detailed images of brain structures, allowing for rapid assessment of acute conditions such as hemorrhage or skull fractures. On the other hand, MRI offers superior soft tissue contrast and is vital for visualizing subtle abnormalities, such as small infarctions or microhemorrhages. The ability of MRI to detect both acute and chronic changes in brain tissue, along with its multiplanar capability, enhances its diagnostic value in neurological disorders. CT and MRI remain indispensable tools due to their high sensitivity, specificity, and non-invasive nature in evaluating brain injuries. The integration of biomarkers measurement may be useful for improving outcomes in neurology diagnostics.

The selection of patients for CT has been suggested to be an important stage in the diagnostic of mild TBI, since it allows detection of serious damage at an early stage, which may change patient management tactics [123]. The authors questioned the need for CT in patients with mild TBI, since 90–95% of the examined patients do not have intracranial lesions and risk unnecessary exposure to radiation [123].

GFAP and UCHL1 or S100B were also able showed results as biomarkers capable to reduce unnecessary CT scanning with high negative predictive value for the absence of intracranial injuries [155]. The advantages of biomarkers also include their ability to work as predictors of the pathological brain image on CT [156]. Thus, biomarkers may have positive aspects as a diagnostic method of brain damage, in combination with or alongside clinical and neuroimaging data [157].

#### COVID-19 and markers of neurological manifestations

Neurological problems have been associated with COVID-19. According to a study conducted in Spain, the most common neurological disorders in patients who were hospitalized for COVID − 19 were anosmia-dysgeusia (44%), headache (44%), myalgia (43%) and dizziness (36%). Additional reports also occurred of encephalopathy (8%), syncope (7%), convulsions (2%), IS (2%) [158]. A retrospective study of neurological manifestations in 613 COVID-19 patients in Brazil reported myalgia (25.6%), headache (22%), fatigue (22%), drowsiness (16%), anosmia (14%), disorientation (8.8%), ageusia (7.3%), convulsions. 2.8%) and dizziness (1.5%). Twelve patients (2%) had strokes (IS: 9) and 149 (24.3%) had encephalopathy. According to the study, elderly patients, patients requiring mechanical ventilation, and patients with encephalopathy are at the highest risk. In a study from China, 78 COVID-19 patients out of 214 (36.4%) had neurological manifestations. Patients with more severe infections also had higher occurrences of neurologic manifestations compared to patients with less severe infections: cerebrovascular disease (5.7% versus 0.8%) and impaired consciousness (14.8% versus 2.4%) [159]. Co-occurrence with viral mRNA and inflammatory markers has been reported in COVID-19 patients. However, predictive models have yet to be developed and tested [160].

## Conclusion

In this wide ranging analysis, we present an overview of 41 biomolecules with potential for use as biomarkers of brain damage. These were identified through database-searches of Pubmed and clinicaltrials.gov. Brain function analysis panels that include protein S100B, NSE, BDNF, PARK7 have demonstrated benefits in clinical trials and recent articles on these markers focus on their application in clinical practice. Clinical assessment appear to perform best when biomarkers are used in conjunction with each other as well as with other clinical parameters. At present, biomarkers do not replace neuroimaging methods, but may help to avoid unnecessary CT-scanning and may show positive aspects as a diagnostic method of brain damage, in combination with or alongside clinical and neuroimaging data. The work related to finding the most optimal combination of biomarkers and parameters for clinical diagnostics is receiving increased attention and the preliminary results are very promising. In recent years, microRNAs have become an increasingly studied topic in the search for new biomarkers, in addition to miRNAs that regulate the expression of neuron-specific proteins. Recent studies also highlight the possibility of assessing different types of brain damage, such as: AIS, TBI, TIA and subarachnoid hemorrhage. Biomarkers are increasingly critical for evaluating new therapeutic approaches including pharmacological treatments. The biological relationship of these biomarker to key pharmacological targets such as such as kinases [161], GPCRs [162], soluble ligands [163], ligand-gated ion channels and other proteins bound to the membrane [164], receptor complexes [165] and other key proteins is important for further studies.

## Data Availability

Information on referenced clinical trials is available to the public at http://clinicaltrials.gov [167].

## Abbreviations

AIS: acute ischemic stroke
AMS: acute minor stroke
aSAH: aneurysmal subarachnoid hemorrhage
AT: III-antithrombin III
AUC: Area under the curve
BBB: blood-brain barrier
BDNF: Brain-Derived Neurotrophic Factor
BNP: Brain natriuretic peptide
CA: cardiac arrest
CKBB: creatine kinase B-type
CLU: clusterin
CNS: central nervous system
COVID: 19-Coronavirus disease 2019
CPSS: Cincinnati Prehospital Stroke Scale
CRP: C-reactive protein
CSF: Cerebrospinal fluid
CT: Computer tomography
DCI: delayed cerebral ischemia
ELISA: enzyme-linked immunosorbent assay
ENO2: Gamma-enolase
FAST: Face arm and speech test
GFAP: Glial fibrillary acidic protein
GFAP: BPD-GFAP breakdown products
GPCRs: G protein-coupled receptors
GRIN2A: Glutamate receptor ionotropic
NMDA 2A, H-FABP: Heart-type fatty acid binding protein
HCY: homocysteine
HDAC2: histone deacetylase 2
HIV: Human Immunodeficiency virus
ICH: intracerebral hemorrhage
ICT: inner cerebral trauma
IFABP: Intestinal Fatty Acid-Binding Protein
IL: 6
17: Interleukins 6 and 17
IMA: ischemia-modified albumin index
IQR: Inter-quartile range
IS: Ischemic stroke
KIF5A: Kinesin heavy chain isoform 5 A
lncRNAs: long non-coding RNA molecules
LVO: large vessel occlusion
MBP: Myelin basic protein
MCAO: Middle Cerebral Artery Occlusion
MCP: 1-monocyte chemoattractant protein 1 (chemokine (C-C motif) ligand 2 (CCL2))
MELAS: mitochondrial encephalomyopathy lactic acidosis and stroke-like episodes
miRNA: microRNA molecules
MMP-2 7, 9, 10, 13 and 16: Matrix metalloproteinases 2, 7, 9, 10, 13 and 16
MMX: Multimarker Index
MOBP: Myelin-associated oligodendrocyte basic protein
MRI: Magnetic resonance imaging
MT: 3-Metallothionein-3
NAD: Nicotinamide adenine dinucleotide
NDKA: Nucleoside diphosphate kinase A
NF: κB-Nuclear factor kappa-light-chain-enhancer of activated B cells
NfH: Neurofilament heavy chain
NfL: Neurofilament light chain
NfM: Neurofilament medium chain
NIHSS: National Institutes of Health Stroke Scale
NLK: Serine/threonine-protein kinase NLK
NPPB: B-type natriuretic peptide
NR2: NR2 subunit of the NMDA receptor
NR2A: Glutamate receptor ionotropic
NMDA 2A, NR2B: Glutamate receptor ionotropic
NMDA 2B, NSE: neuron-specific enolase
OPALIN: Oligodendrocytic myelin paranodal and inner loop protein
PAI1: Plasminogen Activator Inhibitor 1
PARK7: Parkinson disease protein 7
PCR: Polymerase chain reaction
PCT: Procalcitonin
PLAT: Tissue-type plasminogen activator
pNfH: phosphorylated axonal neurofilament subunit H
PTM: posttranslational modifications
qRT: PCR-quantitative reverse transcription PCR
RNAs: Ribonucleic acid
RPS6KA2: AS-Ribosomal Protein S6 Kinase A2 antisense oligonucleotide
S100B: Protein S100-B
SAA1: Serum Amyloid A1
SBI: silent brain infarction
SIRT2: Sirtuin 2
SNAP: 25-Synaptosomal-associated protein 25
sNF: serum Neurofilaments
SNTF: αII-spectrin N-terminal fragment
T3: triiodothyronine
T4: thyroxine
tau: Microtubule-associated protein tau
TBI: traumatic brain injury
Tg: thyroglobulin
TIA: transient ischemic attack
TLDA: TaqMan Low Density Array
TSH: thyroid-stimulating hormone
TSP: triage stroke panel
UCH: L1-Ubiquitin carboxyl-terminal hydrolase isozyme L1
UFD1: Ubiquitin Fusion Degradation Protein-1
vWF: von Willebrand factor
WHO: World Health Organization
